# Cross-Domain Recommendation Based on Sentiment Analysis and Latent Feature Mapping

**DOI:** 10.3390/e22040473

**Published:** 2020-04-20

**Authors:** Yongpeng Wang, Hong Yu, Guoyin Wang, Yongfang Xie

**Affiliations:** 1 Chongqing Key Laboratory of Computational Intelligence, Chongqing University of Posts and Telecommunications, Chongqing 400065, China; wangyp946@foxmail.com (Y.W.); wanggy@cqupt.edu.cn (G.W.); 2 School of Information Science and Engineering, Central South University, Changsha 410083, China; yfxie@csu.edu.cn

**Keywords:** cross-domain recommendation, sentiment analysis, latent sentiment review feature, non-linear mapping

## Abstract

Cross-domain recommendation is a promising solution in recommendation systems by using relatively rich information from the source domain to improve the recommendation accuracy of the target domain. Most of the existing methods consider the rating information of users in different domains, the label information of users and items and the review information of users on items. However, they do not effectively use the latent sentiment information to find the accurate mapping of latent features in reviews between domains. User reviews usually include user’s subjective views, which can reflect the user’s preferences and sentiment tendencies to various attributes of the items. Therefore, in order to solve the cold-start problem in the recommendation process, this paper proposes a cross-domain recommendation algorithm (CDR-SAFM) based on sentiment analysis and latent feature mapping by combining the sentiment information implicit in user reviews in different domains. Different from previous sentiment research, this paper divides sentiment into three categories based on three-way decision ideas—namely, positive, negative and neutral—by conducting sentiment analysis on user review information. Furthermore, the Latent Dirichlet Allocation (LDA) is used to model the user’s semantic orientation to generate the latent sentiment review features. Moreover, the Multilayer Perceptron (MLP) is used to obtain the cross domain non-linear mapping function to transfer the user’s sentiment review features. Finally, this paper proves the effectiveness of the proposed CDR-SAFM framework by comparing it with existing recommendation algorithms in a cross-domain scenario on the Amazon dataset.

## 1. Introduction

A recommendation system helps a user discover the information he/she wants such as products and content from the massive information produced by the Internet. Recommendation systems are primarily used in commercial applications. A recommendation system helps users find valuable information as the interested information can be recommended to users. This is a win-win situation for both consumers and manufacturers. A good recommendation system can not only accurately detect the user’s behavior, but also help users find the potential information they are interested in.

There are a lots of achievements in recommendation systems, which try to enhance the accuracy, diversity and novelty of recommendation. For example, a collaborative filtering-based recommendation algorithm [1] is one of the most popular and widely used algorithms and it can be divided into two categories—user-based recommendations and item-based recommendations. Among model-based collaborative filtering methods, matrix factorization [2] technology is popular because it is extremely scalable and easy to implement. The accuracy of the matrix factorization recommendation to a great extent depends on the rating matrix. However, in real life, with the rapid growth of users and items, the rating matrix is very sparse, which has a great impact on the recommendation of new users and new items. Thus, the cold-start and data sparsity problems in the recommendation process arise.

Recently, more and more researchers [3] have researched cross-domain recommendation by introducing the concepts of source domain and target domain in order to solve the problem of data sparsity and cold-start in the single-domain recommendation process. The purpose of cross-domain based recommendation is to use the richer information in multiple domains than in a single-domain and to transfer the knowledge between different domains effectively based on the idea of transfer learning. One of the key assumptions that cross-domain recommendation can work is that there exist consistency or correlation between users’ interest preferences or item features between domains. This hypothesis is also supported by some research work. The cross-domain recommendation utilizes the consistency or correlation between domains, such as the intersection of users and items, the similarity between user interests, the similarity between item features, and the relationship between latent factors, and so forth, to make up for the problem of insufficient information in the target domain.

However, the existing cross-domain recommendation methods are only based on the sharing and transfer of knowledge in the text information such as rating, tag or review, and ignore the latent sentiment information in the review. User reviews usually include user’s subjective views, which can reflect the user’s preferences and sentiment tendencies to various attributes of the item. Fully mining and using the implied sentiment information is helpful to solve the cold-start problems and data sparsity in the process of cross-domain recommendation. The existing cross-domain recommendation algorithms using user reviews do not make full use of the sentiment information in these reviews. They mixed positive sentiment, neutral sentiment and negative sentiment together to realize knowledge transfer, which will weaken or even lose some sentiment information of users, especially negative sentiment. Therefore, it has a great significance to make cross-domain recommendations by combining the user’s sentimental features implicit in the review information.

To address the problem of cold-start in the process of recommendation, we propose a cross-domain recommendation algorithm based on sentiment analysis and latent feature mapping (shorted by CDR-SAFM) in this paper, by combining with the implicit sentiment information in user reviews. First, this paper divides the sentiment of user review information into three categories based on the theory of three-way decisions [4,5], namely positive, negative and neutral. Then, the Latent Dirichlet Allocation method is used to model users’ semantic orientation to generate users’ latent sentiment review features. Finally, the Multi-Layer Perceptron method is used to obtain the cross-domain non-linear mapping function to transfer the user’s sentiment review features. The main contributions are concluded as follows:

- A novel algorithm for cross-domain recommendation named CDR-SAFM is proposed for cold-start users in target domain. It employs sentiment analysis and latent feature mapping and it can transfer latent sentiment review feature from source domain to target domain and make recommendation for cold-start users in target domain.
- Basing on the idea of three-way decisions, we take into account neutral sentiment to generate the latent sentiment review feature from both the source and target domains, which can affect ratings in the two domains.
- The LDA models is used to generate user latent review features. When generating features, we consider the sentiment information from reviews, and generate the user sentiment review features in different domains.
- The Multi-layer Perceptron is employed to accurately map the latent sentiment review feature from the source domain to the target domain, which improves recommendation accuracy.

The rest of the paper is organized as follows. Section 2 introduces the related work. In Section 3, the preliminary work is reviewed. A detailed description of our algorithm is stated in Section 4. Subsequently, in Section 5, we discuss experimental settings and the comparative results. Finally, we conclude the paper in Section 6.

## 2. Related Work

Aiming at the cold-start problem caused by too sparse rating matrices in different domains, some scholars [6,7,8] tried to use the common users in the two domains as bridges, using the data in the auxiliary domain to solve the cold start problem in the target domain by feature mapping. Pan and Yang [6] learned a transformation matrix based on the feature representation of common users in the two domains, and realized the mapping of features between different domains. The transformation matrix implements a linear mapping, and the mapping relationship of features in different domains may be non-linear. Then, Xin et al. [7] modeled a non-linear feature mapping function through a multi-layer perceptron, and obtained a better mapping effect than the transformation matrix. Wei et al. [8] implemented the recommendation of e-commerce website products to social networking sites through common users and the recommendations for cold-start users are made through the mapping of user features.

Moreover, in order to make full use of the hidden user and item relationships between domains, some works [9,10,11] proposed combining them with transfer learning. For example, Jiang et al. [9] connected different domains with each other through social networks, forming a hybrid graph with a social network-centric star structure, and used a random walk algorithm to predict the user and item relationship. In addition, some scholars [12,13,14] try to analyze the behavior of users in multiple social web platforms. The semantic relationships of items in each domain are also used for knowledge transfer. Yang et al. [15] introduced the tag system into the cross-domain recommendation, and successfully implemented the cold-start problem of recommending, that is, to recommend movies to the new user based on the blog posts on Weibo. The basic idea of the work is to use the semantic relationship between tags on user blog posts and movie tags as a bridge to associate users with movies, and then it predicts user preferences based on graph models. Shi et al. [16] proposed a cross-domain recommendation algorithm for collaborative filtering with fused labels. The model first uses the rich label information in the labeling system that the user has labeled the item to calculate the user-user similarity matrix and the item-item similarity matrix. Then it uses the information as a smoothing term to improve the probability matrix decomposition model PMF [17]. The trained user and item feature vectors can also satisfy the similarity relationship between users and items on the basis of minimizing the error between the predicted rating and the actual rating. Kumar et al. [18] used the Latent Direchlet Allocation (LDA) topic model [19] to model the user’s tagging information to build a user feature topic sharing space shared by different domains and then, based on this space, to find users with similar preferences in different domains and implement cross-domain recommendation.

Furthermore, Song et al. [20] believed that, compared with the rating information, the user review information cannot only express the user’s preferences for the item, but also cover other user interest preferences. Therefore, they proposed a joint tensor decomposition model based on review information for cross-domain recommendation. The model is trained by using the AIRS method rating information proposed in Reference [21], analyzing user reviews from multiple different angles, and obtaining the user’s rating and degree of interest at each angle. This is achieved by sharing feature vectors in the source and target domain Knowledge transfer. Hu et al. [22] aimed at the problem of data sparseness and integrated auxiliary information such as product reviews and news headlines to form a hybrid filtering method transferring knowledge from other source domains, such as improving movie recommendations with knowledge in the book domain, and thus forming a transfer of learning methods.

## 3. Preliminary Work

### 3.1. Sentiment Analysis

Text sentiment analysis refers to the process of analyzing, processing, and extracting subjective text with emotion using natural language processing and text mining technology [23]. The sentiment analysis task can be divided into chapter level, sentence level, word or phrase level according to its analysis granularity; according to its processing text category, it can be divided into sentiment analysis based on product reviews and sentiment analysis based on news reviews; according to its research tasks types can be divided into sub-problems such as sentiment classification, sentiment retrieval and sentiment extraction.

Sentiment classification refers to the identification of subjective text in a given text, whether it is positive or negative, which is the most researched in the field of sentiment analysis. There are usually a lot of subjective texts and objective texts in network texts. Objectivity text is an objective description of things, without emotion color and emotional tendency, and subjective text is the author’s views or ideas on various things, with emotional tendencies such as the author’s likes and dislikes. The object of sentiment classification is subjective text with emotional tendency, so emotion classification must first be subjective and objective classification of text. The subjective and objective classification of texts is mainly based on the recognition of sentiment words. Using different text feature representation methods and classifiers for classification, subjective and objective classification of web texts in advance can improve the speed and accuracy of sentiment classification. Looking at the current research work on subjective text sentiment analysis, the main research ideas are divided into semantic-based sentiment dictionary methods and machine learning-based methods.

In the semantic-based sentiment dictionary method, the construction of sentiment dictionary is the premise and basis of sentiment classification. At present, it can be classified into four categories: general sentiment words, degree adverbs, negative words, and domain words. The construction method of emotional dictionaries is to use existing electronic dictionary extensions to generate emotional dictionaries. English is mainly based on the expansion of the English dictionary WordNet to form SentiWordNet lexicon. Hu and Liu [24] have manually established the seed adjective vocabulary and used the synonymous relationship between words in WordNet to determine the emotion tendency of emotion words, and use this to judge the emotional polarity of the point of view.

The tendency calculation of semantic-based sentiment dictionaries is different from the machine learning algorithms that require a large number of training datasets. It mainly analyzes the special structure and sentiment tendency words of text sentences by using sentiment dictionary and sentence lexicon, and uses weight algorithm instead of traditional manual discrimination or simply statistical method for sentiment classification. Emotional words with different sentiment intensity are assigned different weights, and then weighted summation is performed.

Finally, the threshold is determined to judge the tendency of the text. In general, the weighted calculation result is positive to indicate positive tendency; the result is negative to indicate negative tendency, and the score is zero to indicate no tendency. Compared with the classification algorithm based on machine learning, the sentiment dictionary-based method is a coarse-grained tendency classification method, but because it does not rely on a well-labeled training set, implementation is relatively simple. It can effectively and quickly classify sentiment for web texts in universal fields.

Sentiment analysis has been widely used in recommendation systems. Calculating the sentiment orientation of user reviews has been studied by some researchers. Diao [25] built a language model component in the JMARS model they proposed to capture hidden points in reviews. Zhang [26] performed phrase-level sentiment analysis on user reviews to extract clear product features and user opinions to generate interpretable recommendation results. Li [27] proposed a SUIT model for sentiment analysis using both text themes and user items. In this article, we apply sentiment analysis to cross-domain recommendation tasks, focusing on finding latent sentiment review features of users and mapping them from the source domain to the target domain.

### 3.2. Topic Model

In natural language processing, the Latent Dirichlet Allocation (LDA) [19] is a powerful and practical tool for analyzing large text documents. Latent Dirichlet Allocation is also a common method to solve the cold start problem. LDA can automatically cluster words into topics and discover relationships between documents from a dataset. It assumes that the authors of the resource have multiple themes; based on their vocabulary, the author chooses specific vocabulary to describe their topic. Formally, resources are distributed on topics and topics are distributed on vocabularies. Vocabulary consists of different words in the corpus.

**Figure 1:**
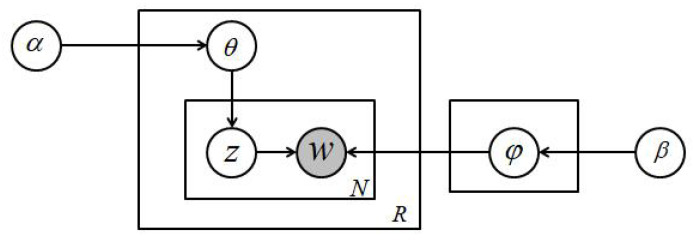
Standard Latent Dirichlet Allocation (LDA).

As shown in Figure 1, the symbolic representation of standard LDA is shown. φ refers to the representation of topic in vocabulary, and θ refers to the distribution of resources across topics. Variables α and β are hyperparameters of the model. Parameter α controls the distribution of resources on topics, and parameter β controls the distribution of topics in vocabulary. Variable *z* represents subject assignment, while variable *w* is the observed word. *R* is the number of resources in the corpus, and *N* is the number of words in the resources. Parameter *K* indicates the number of topics suitable for corpus. *K* is allocated during initialization. Among the variables, only *w* is the observation variable and the rest is the latent variable.

The process of LDA starts from the sampling of topic *z*. Based on topic *z*, the word *w* is obtained by sampling φ with a polynomial, which is described as follows (1)–(5):

(1) $$\varphi \left(w_{i}|k,\beta \right)=\frac{n(w_{i},k)+\beta }{\sum_{w\in V}n(w,k)+(\beta -1)}.$$

(2) $$\theta \left(k|d,\alpha \right)=\frac{n(r,k)+\alpha }{\sum_{k\in K}n(r,k)+(\alpha -1)}.$$

Variable *k* represents the *k* topic of model sampling, and $\varphi (w_{i}|k,\beta )$ calculates the probability that word $w_{i}$ is the *k* topic in the dictionary. $n(w_{i},k)$ indicates the number of occurrences of word $w_{i}$ assigned to topic *k*. θ(k|d,α) calculates the probability of document *d* for topic *k*. n(r,k) is the number of times resource *r* is assigned to topic *k*. The joint distribution of the model is as follows:

(3) $$p\left(\theta ,z,w|\alpha ,\beta \right)=p\left(\theta |\alpha \right)\prod_{n=1}^{N}p(z_{n}|\theta )p\left(w_{n}|z_{n},\beta \right).$$

(4) p(θ,z|w,α,β)=p(θ,z,w|α,β)p(w|α,β).

Equation (4) is used to infer markers, but it is difficult to calculate the real posterior distribution. Gibbs sampling is used to estimate the posterior distribution in order to deal with the difficulty. Gibbs sampling starts by randomly assigning a word to a topic. In subsequent iterations, it assigns a word to a topic based on the following equation:

(5) $$p\left(z_{i}=k|z_{-i},w,\theta \right)=\frac{n{\left(w_{i},k\right)}_{-i}+\beta }{\sum_{w\in V}n(w,k)+(\beta -1)}\times \frac{n{\left(r,k\right)}_{-i}+\alpha }{\sum_{k\in K}n(r,k)+(\alpha -1)}.$$

where $n{\left(w_{i},k\right)}_{-i}$ represents the number of times a word $w_{i}$ appears in topic *k* without including the currently assigned task.

## 4. The CDR-SAFM Algorithm

### 4.1. Notations

We suppose that there are two domains sharing the same user. Users who appear in one domain can appear in another domain. In this sense, the two domains share the same user. Without losing generality, one domain is called the source domain. The other is called the target domain.

Define the recommended objects in the target domain as items. Let $U=\{u_{1},u_{2},\ldots ,u_{\left|U\right|}\}$ represent the common users of source domain and target domain, that is overlapping users. Let $J_{S}=\left\{i_{1},i_{2},\ldots ,i_{|J_{S}|}\right\}$ and $J_{T}=\left\{l_{1},l_{2},\ldots ,l_{|J_{T}|}\right\}$ be the item sets from the source and target domains respectively. The user review dataset is represented as $SR_{U}=\left\{r_{u_{1}},r_{u_{2}},\ldots ,r_{u_{\left|U\right|}}\right\}$ in source domain and $TR_{U}=\left\{r_{u_{1}},r_{u_{2}},\ldots ,r_{u_{\left|U\right|}}\right\}$ in target domain, where $r_{u_{i}}$ is all of reviews of user $u_{i}$ in the corresponding domain. Similarly, we let $TR_{I}=\left\{r_{i_{1}},r_{i_{2}},\ldots ,r_{i_{|J_{T}|}}\right\}$ denote the item review dataset in target, where $r_{i_{j}}$ is all of reviews which item $i_{j}$ acquired in target domain. $R^{s}$ and $R^{t}$ be two rating matrices from the source and target domains respectively, where $R_{ij}^{s}$ is the rating that user $u_{i}$ gives to item $i_{j}$ in the source domain and $R_{ij}^{t}$ is the corresponding rating in the target domain.

### 4.2. Problem Formulation

**Figure 2:**
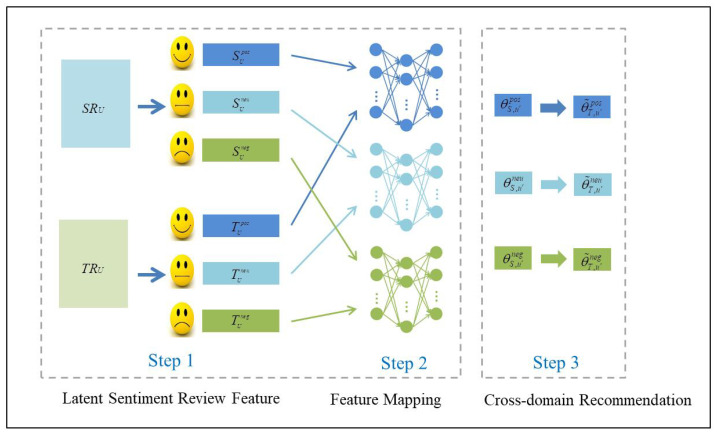
Illustrative diagram of the CDR-SAFM algorithm.

Given the review information $SR_{U}$ and $TR_{U}$ of two domains, and overlapping user sets *U* across domains, we aim to analyze the sentiment information of the source domain and the target domain, use the common user as a bridge to realize the knowledge transfer from the source domain to the target domain, and solve the problem of rating prediction of cold-start users in the target domain. For this purpose, we propose a cross-domain recommendation algorithm, CDR-SAFM, based on sentiment analysis and latent feature mapping. This framework contains three major steps, that is, latent sentiment review features modeling, latent sentiment review features mapping and cross-domain recommendation, as illustrated in Figure 2.

At the first step, we aim to analyze the emotional orientation of user reviews in two domains through sentiment analysis methods, so that the original dataset in the two domains is divided into three parts: positive reviews, neutral reviews, and negative reviews. Reviews in the source domain are divided into $SR_{U}^{pos}$, $SR_{U}^{neu}$ and $SR_{U}^{neg}$, and reviews in the target domain are divided into $TR_{U}^{pos}$, $TR_{U}^{neu}$ and $TR_{U}^{neg}$. Then we aim to find the representation of latent sentiment review features by LDA, including positive sentiment review features, neutral sentiment review features, and negative sentiment review features. The latent sentiment review feature assumes the association between a user’s reviews on an item, and the user’s reviews on an item are actually the result of the combination of the user and the sentiment review features. That is to say, users’ reviews always contain sentiment features. In order to reduce the influence of previous sentiment classification methods on the overall algorithm results, we classify users’ review information by users’ rating of the item, because a user’s review emotion polarity of an item can be reflected in the rating information. For example, if a user likes an item, the user’s final reviews will be more positive, and the user’s rating will be higher.

In the second step, we aim to obtain a mapping function for modeling cross-domain relationship of sentiment review features. We assume that there is a latent mapping relationship between the sentiment review features from source domain and target domain, and then capture this relationship by mapping function. To avoid the mutual interference among the features of positive, neutral and negative sentiment review during the process of knowledge transfer, we use mapping function to model the cross-domain relationship of different sentiments respectively. In order to avoid the lack of different sentiment review features of users in the mapping process, we train the mapping function of different emotions by preprocessing the data and using the common users in the two domains whose sentiment review features are not missing.

Finally, we recommend a cold-start user in the target domain. Using this method, we can get the corresponding latent sentiment review feature in the target domain and use these features to affect the final recommendation results. Different mapping results of sentiment review features of cold-start users have different influence on users’ ratings. Therefore, we set different weights for different results of sentiment review features to get the emotional ratings of cold-start users. The complete CDR-SAFM algorithm is presented in Algorithm 1.
**Algorithm 1:** The CDR-SAFM algorithm.**Require:**Source domain $SR_{U}$, target domain $TR_{U}$;Common User set *U*;**Ensure:**  Make recommendation for cold-start users in the target domain; ** Sentiment Analysis**1:Learn $\left\{SR_{U}^{pos},SR_{U}^{neu},SR_{U}^{neg}\right\}$ from $SR_{U}$;2:Learn $\left\{TR_{U}^{pos},TR_{U}^{neu},TR_{U}^{neg}\right\}$ from $TR_{U}$; **Latent sentiment review feature**3:Learn $\left\{S_{U}^{pos},S_{U}^{neu},S_{U}^{neg}\right\}$ from $\left\{SR_{U}^{pos},SR_{U}^{neu};,SR_{U}^{neg}\right\}$;4:Learn $\left\{T_{U}^{pos},T_{U}^{neu},T_{U}^{neg}\right\}$ from $\left\{TR_{U}^{pos},TR_{U}^{neu};,TR_{U}^{neg}\right\}$; **Latent Sentiment Review Feature Mapping**5:Learn the mapping function $f_{pos}\left(\cdot \right)$, $f_{neu}\left(\cdot \right)$ and $f_{neg}\left(\cdot \right)$ by users across domain; **Cross-domain Recommendation**6:Get affine features $U_{new}^{T.pos}$, $U_{new}^{T.neu}$ and $U_{new}^{T,neg}$ of target users;7:Make recommendation for target users.

### 4.3. Latent Sentiment Review Feature Modeling

**Figure 3:**
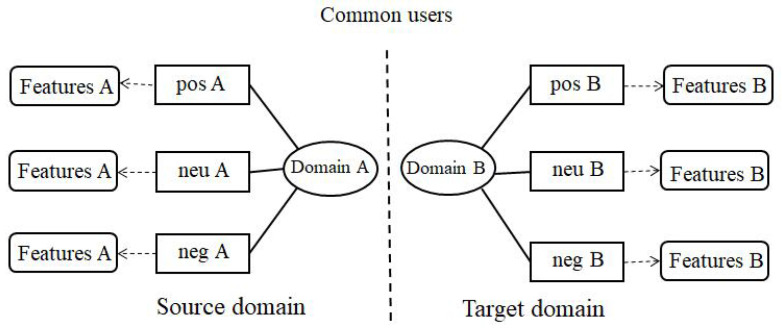
Sentiment analysis and feature extraction.

In this section, we aim to analyze the sentiment information hidden in the user’s review information and extract the user’s review features under different sentiments from the source domain and the target domain, as illustrated in Figure 3.

#### 4.3.1. Sentiment Analysis

According to general psychology, sentiment has an important influence on one’s behavior and choices. Sentiment analysis plays an important role in information retrieval. It clarifies people’s thoughts and feelings about something or someone in a certain situation. This kind of high level information can be used in many applications, such as customer review analysis, business and government intelligence, personalized recommendation and so on. User reviews on online platforms show similar sentiment expressions, which is generated by similar psychological stimulation. Therefore, it is valuable to combine the latent sentiment information in reviews with cross domain recommendation.

Sentiment analysis (SA) is a process of analyzing, processing, summarizing and reasoning subjective characters with emotional color. Among them, sentiment analysis can also be divided into emotional orientation analysis, emotional level analysis, subjective and objective analysis, and so forth. The purpose of emotional orientation analysis is to judge the positive, negative and neutral meaning of the text. In most application scenarios, there are only two types. For example, the two words “love” and “disgust” belong to different emotional orientations.

However, in the past work based on sentiment analysis, most of the sentiment analysis problems are expressed as—given a set of review *R*, a sentiment classification algorithm can divide each sentence of a review r∈R into two categories—positive $R^{pos}$ and negative $R^{neg}$. In real life, some texts can not be directly classified into positive sentiment or negative sentiment. Therefore, in this paper, based on the three-way decision ideas, we divide the source domain review data information into positive $SR_{U}^{pos}$, neutral $SR_{U}^{neu}$ and negative $SR_{U}^{neg}$, and the target domain review data into $TR_{U}^{pos}$, $TR_{U}^{neu}$ and $TR_{U}^{neg}$. Because the sentiment analysis algorithm is not the focus of this paper, it is suggested that readers refer to the relevant literature. To achieve this goal, we use a method based on statistics to do sentiment analysis on the review dataset.

We extract “ratings” and “reviews” from the dataset for analysis. “Ratings” represents the user rating of the item in the review, and the rating range is 1–5. “reviews” is the text information of the user review. We randomly selected 1000 reviews each of 1–5, and performed basic de-punctuation, lowercase, and stop word processing on the text to count the word frequency to make a word cloud diagram. Based on the analysis of word cloud diagram with different ratings, most reviews rated as 1, 2 are negative sentiment reviews, and reviews rated as 4, 5 are mostly positive sentiment reviews. However, it is difficult to define the division of reviews with a score of 3, which also proves the idea of three-way decision ideas, so we regard them as a separate category. Therefore, we can divide the dataset into positive sentiment reviews with a rating of 4 or 5, neutral sentiment reviews with a rating of 3 and negative sentiment reviews with a rating of 1 or 2.

#### 4.3.2. Latent Sentiment Review Feature

Obtaining the latent sentiment feature of users is the key for improving the performance of recommendation algorithms. Review-based recommendation algorithms tend to extract latent feature by topic models. In this paper, we use an LDA topic model to extract latent feature. Each latent feature (topic) extracted by LDA is associated with a set of keywords. Thus, we can get interpretable recommendation results by matching the topic distribution of users and items.

Take the analysis of user features under positive sentiment in source domain as an example (other similar). We use the review information of all users as a corpus for LDA model training, and use all the reviews of each user as a document. Finally, we find the word distribution of the topics and the topic distribution of the document under the positive sentiment of the source domain. The topic distribution of the document is the user’s latent sentiment review feature, which represented as $S_{U}^{pos}=\left\{\theta_{S,u_{1}}^{pos},\theta_{S,u_{2}}^{pos},\ldots ,\theta_{S,u_{\left|U\right|}}^{pos}\right\}$. Similarly, we can calculate the latent sentiment review feature of users in the source domain and target domain under the positive, negative and neutral sentiment. The sentiment review features in the source domain are $S_{U}^{pos}=\left\{\theta_{S,u_{1}}^{pos},\theta_{S,u_{2}}^{pos},\ldots ,\theta_{S,u_{\left|U\right|}}^{pos}\right\}$, $S_{U}^{neu}=\left\{\theta_{S,u_{1}}^{neu},\theta_{S,u_{2}}^{neu},\ldots ,\theta_{S,u_{\left|U\right|}}^{neu}\right\}$ and $S_{U}^{neg}=\left\{\theta_{S,u_{1}}^{neg},\theta_{S,u_{2}}^{neg},\ldots ,\theta_{S,u_{\left|U\right|}}^{neg}\right\}$, where $\theta_{S,u_{i}}^{pos}$ represent positive review features of user $u_{i}$ in the source domain. Similarly, $\theta_{S,u_{i}}^{neu}$ and $\theta_{S,u_{i}}^{neg}$ represent the neutral review features and the negative review features, respectively. The sentiment review features in the target domain are $T_{U}^{pos}=\left\{\theta_{T,u_{1}}^{pos},\theta_{T,u_{2}}^{pos},\ldots ,\theta_{T,u_{\left|U\right|}}^{pos}\right\}$, $T_{U}^{neu}=\left\{\theta_{T,u_{1}}^{neu},\theta_{T,u_{2}}^{neu},\ldots ,\theta_{T,u_{\left|U\right|}}^{neu}\right\}$ and $T_{U}^{neg}=\left\{\theta_{T,u_{1}}^{neg},\theta_{T,u_{2}}^{neg},\ldots ,\theta_{T,u_{\left|U\right|}}^{neg}\right\}$, where $\theta_{T,u_{i}}^{pos}$ represent positive review features of user $u_{i}$ in the target domain. $\theta_{T,u_{i}}^{neu}$ and $\theta_{T,u_{i}}^{neg}$ represent the neutral review features and the negative review features, respectively. In addition, $T_{I}^{pos}=\left\{\theta_{T,l_{1}}^{pos},\theta_{T,l_{2}}^{pos},\ldots ,\theta_{T,l_{\left|I\right|}}^{pos}\right\}$, $T_{I}^{neu}=\left\{\theta_{T,l_{1}}^{neu},\theta_{T,l_{2}}^{neu},\ldots ,\theta_{T,l_{\left|I\right|}}^{neu}\right\}$ and $T_{I}^{neg}=\left\{\theta_{T,l_{1}}^{neg},\theta_{T,l_{2}}^{neg},\ldots ,\theta_{T,l_{\left|I\right|}}^{neg}\right\}$ respectively represent the distribution of sentiment review topics of all items in the target domain, and $\theta_{T,l_{i}}^{pos}$ represents the distribution of positive review topics of items $l_{i}$ in the target domain.

### 4.4. Sentiment Review Feature Mapping

How to transfer users’ sentiment information effectively is an important problem to be solved in this paper. Existing cross-domain recommendation algorithms that use user reviews do not make full use of the sentiment information in these reviews. They achieve knowledge transfer by mixing positive, neutral, and negative sentiment, which will weaken or even lose some of the user’s sentiment Information, especially negative sentiment. For example, users may be very concerned about the plot of a novel and make positive reviews on the plots of some novels in the field of e-books (source domain), while making negative reviews on the plots of other novels. If we transfer the knowledge obtained from user reviews from the source domain to the target domain without distinguishing the emotional orientation of these reviews, the underlying sentiment factors in the reviews will be mixed together and transferred to the target domain as user features. In movies (target domain), movies with poor plots will match user features transferred to the target domain. But users may not like the movie.

**Figure 4:**
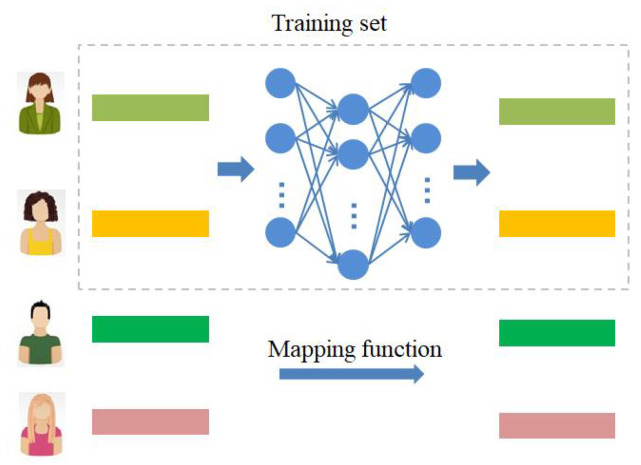
Mapping architecture.

To connect the source domain and the target domain, we assume that we can get the relationship between domains through a mapping function. The mapping architecture is shown in Figure 4.

To obtain the mapping function, we can formalize the learning process into a supervised regression problem. In particular, we minimize the mapping loss to obtain the mapping function.

(6) $$\min_{\theta }\sum_{ui\in U}L\left(f_{U}\left(U_{i}^{s};\theta \right),U_{i}^{t}\right),$$

where L(·,·) is the loss function that defines the corresponding vector in the source domain and the target domain. Because the input and output of the mapping function are multi-dimensional numerical vectors, we choose the square error as the loss function.

Multilayer Perceptron (MLP) is a nonlinear transformation, which is more flexible than a linear mapping function. Thus, we choose mapping based on multi-layer perceptron to realize cross domain connection between different domains. It makes the user’s sentiment review feature in the source domain play a complementary and perfect role in the target domain recommendation.

MLP can be optimized using back propagation. The optimization problem can be formalized as

(7) $$\min_{\theta }\sum_{u_{i}\in U}L\left(f_{mlp}\left(U_{i}^{s};\theta \right),U_{i}^{t}\right),$$

where $f_{mlp}\left(\cdot ;\theta \right)$ is MLP mapping function, θ is its parameter set, which are the weight matrices and bias terms between layers. In this paper, we use the method of random gradient descent to study its parameters and get MLP mapping function. By traversing the training set, refresh the parameters of MLP with any user $u_{i}$ across the source and target domains. The gradient of parameters is calculated by back propagation algorithm. Until the model converges, the iterative process stops.

In this paper, through sentiment analysis and feature extraction of the source domain and target domain datasets, we can get the user sentiment review features $S_{U}^{pos}$, $S_{U}^{neu}$ and $S_{U}^{neg}$ in the source domain, and the user sentiment review features $T_{U}^{pos}$, $T_{U}^{neu}$ and $T_{U}^{neg}$ in the target domain. In order to connect the source domain and the target domain, we train the feature mapping relationship $f_{MLP}^{pos}$ of positive sentiment in different domains through $S_{U}^{pos}$ and $T_{U}^{pos}$ (the negative and neutral feature mapping relationships are similar, respectively $f_{MLP}^{neu}$ and $f_{MLP}^{neg}$).

Let $S_{U}^{pos}=\left\{\theta_{1}^{S},\theta_{2}^{S},\ldots ,\theta_{N}^{S}\right\}$ denote s set of sentiment review feature in the source domain, $T_{U}^{pos}=\left\{\theta_{1}^{T},\theta_{2}^{T},\ldots ,\theta_{N}^{T}\right\}$ denote s set of sentiment review feature in the target domain. *N* is the number of common users. We formalized mapping problem as: given *N* training samples $\left(\theta_{i}^{S},\theta_{i}^{T}\right)$, $\theta_{i}^{S},\theta_{i}^{T}\in R^{M}$(i=1,2,…,N), we aim to learn a mapping function so that we can get the sentiment review features in the target domain through the sentiment review features in the source domain.

That is to say, $S_{U}^{pos}$ and $T_{U}^{pos}$ are used to train the mapping function of source domain and target domain in positive sentiment review feature. Similarly, $S_{U}^{neu}$ and $T_{U}^{neu}$ are used to train the mapping function of neutral sentiment review feature, and $S_{U}^{neg}$ and $T_{U}^{neg}$ are used to train the mapping function of negative sentiment review features. Specifically, given positive sentiment review features $S_{u^{\prime }}^{pos}$ and $T_{u^{\prime }}^{pos}$ in two domains of user $u^{\prime }$, we use a mapping function f(·;θ) to capture the cross domain relationship $T_{u^{\prime }}^{pos}=f_{pos}\left(S_{u^{\prime }}^{pos};\theta \right)$, where θ is the parameter of the mapping function. Similarly, we can get $T_{u^{\prime }}^{neu}=f_{neu}\left(S_{u^{\prime }}^{neu};\theta \right)$ and $T_{u^{\prime }}^{neg}=f_{neg}\left(S_{u^{\prime }}^{neg};\theta \right)$.

### 4.5. Cross-Domain Recommendation

Given a cold-start user, we cannot recommend it directly because of its sparse data. We first can get the user’s sentiment review feature in the source domain and get the sentiment review feature in the target domain through the learned sentiment feature mapping function. Then by combining the existing user benchmark recommendation results and the influence of sentiment information on user recommendations, we can get the final recommendation results. The specific formal description is as follows:

Given a cold-start user $u^{\prime }$ in the target domain, we classify all the user’s review information into $S_{u^{\prime }}^{pos}$, $S_{u^{\prime }}^{neu}$ and $S_{u^{\prime }}^{neg}$ in the source domain. We can obtain the sentiment review features $\theta_{S,u^{\prime }}^{pos}$, $\theta_{S,u^{\prime }}^{neu}$ and $\theta_{S,u^{\prime }}^{neg}$ by Latent Dirichlet Allocation. Then we aim to get sentiment review features ${\tilde{\theta }}_{T,u^{\prime }}^{pos}$, ${\tilde{\theta }}_{T,u^{\prime }}^{neu}$ and ${\tilde{\theta }}_{T,u^{\prime }}^{neg}$ of user $u^{\prime }$ in the target domain is defined as:

(8) $${\tilde{\theta }}_{T,u^{\prime }}^{pos}=f_{MLP}^{pos}\left(\theta_{S,u^{\prime }}^{pos};\theta pos\right),$$

(9) $${\tilde{\theta }}_{T,u^{\prime }}^{neu}=f_{MLP}^{neu}\left(\theta_{S,u^{\prime }}^{neu};\theta neu\right),$$

(10) $${\tilde{\theta }}_{T,u^{\prime }}^{neg}=f_{MLP}^{neg}\left(\theta_{S,u^{\prime }}^{neg};\theta neg\right).$$

Calculating the topic similarity under the same sentiment in different domains is defined as:

(11) $$SIM^{pos}=\left\{sim_{i,j}^{pos}\right\},i,j=1,2,\ldots ,M,$$

(12) $$SIM^{neu}=\left\{sim_{i,j}^{neu}\right\},i,j=1,2,\ldots ,M,$$

(13) $$SIM^{neg}=\left\{sim_{i,j}^{neg}\right\},i,j=1,2,\ldots ,M.$$

Therefore, we can get the predicted sentiment rating of cold-start users. That is, the impact of sentiment information in user reviews on the final recommendation is defined as:

(14) $$e\left(u^{\prime }\right)=w_{1}\times {\tilde{\theta }}_{T,u^{\prime }}^{pos}\cdot SIM^{pos}\cdot T_{I}^{posT}-w_{2}\times {\tilde{\theta }}_{T,u^{\prime }}^{neg}\cdot SIM^{neg}\cdot T_{I}^{negT}+w_{3}\times {\tilde{\theta }}_{T,u^{\prime }}^{neu}\cdot SIM^{neu}\cdot T_{I}^{neuT},$$

where the parameters $w_{1}$, $w_{2}$ and $w_{3}$ represent different weights. Finally, combined with the user’s benchmark rating and prediction sentiment rating, we can get the final prediction rating of cold-start user $u^{\prime }$ on the item $l_{i}$, which can be expressed as:

(15) $$pre\left(u^{\prime }\right)=R\_base+e\left(u^{\prime }\right),$$

where $R\_base=b_{T}+b_{u^{\prime }}+b_{I_{j}}$, $b_{T}$ represents the average rating of all items in the target domain. The parameter $b_{u^{\prime }}$ is the user rating bias in the source domain and $b_{I_{j}}$ is the item rating bias in the target domain.

## 5. Experiments

In this section, we test the CDR-SAFM algorithm proposed in this paper with a real-world dataset. Firstly, the experimental dataset is introduced, and the possible rating of cold-start users in the target domain is predicted by using review data from two different domains—Electronics, and Movies and TV. Then we compare the proposed model with the common methods in the recommendation system. Finally, we randomly select different percentages of cold-start users and analyze the experimental results.

### 5.1. Experimental Settings

**Datasets.** We employ Amazon cross-domain dataset [28] in our experiment. This dataset contains product reviews and star ratings with 5-star scale from Amazon, including 142.8 million reviews spanning May 1996–July 2014. In our experiment, we select the top two domains with the most widely used in previous studies to employ in our cross-domain experiment: Electronics and Movies & TV. The global statistics of two domains used in our experiment are shown in Table 1.

**Experimental Settings.** In the experiment, we set Electronics as the source domain and Movies & TV as the target domain. After data preprocessing, the number of common users in the two domains is 2406. The items in these two domains are very different, forming a cross domain user sharing scenario. To evaluate the validity and efficiency of the proposed algorithm on cross-domain recommendation task, we randomly remove all the rating information of a fraction of entities in the target domain and take them as cross-domain cold-start entities for making recommendation. For the sake of stringency of the experiments, we set different fraction for cold-start entities, namely, 20%, 50% and 70%. In addition, since different sets of cold-start entities may affect the final recommendation results, we repeatedly sample users for 10 times to generate different sets. Dimension K of latent sentiment review features is set as 50 and 100. For the MLP mapping function, we choose the structure of the MLP as one-hidden layer, the dimension of input and output of the MLP is set as K, whilst the number of nodes in the hidden layer is set as 2K. The weight and bias parameters of the MLP is initialized according to the rule in Reference [29]. Finally, a tan-sigmoid function is employed as the activation function. In order to obtain the mapping function of MLP, we use stochastic gradient descent to learn the parameters. Through the loop on the training set, the parameters of the MLP are updated. This back-propagation algorithm is used to calculate the gradient of the parameters, and this process continues until the model converges.

**Models for Comparison.** We compare the CDR-SAFM algorithm with the following baseline models and algorithms for validating the performance.

- **AVE**: It predicts ratings by the following equation: $r_{ui}=b_{t}+b_{u}+b_{i}$ where $b_{t}$ is the overall average ratings of all items in the target domain, $b_{u}$ denotes the user rating bias in the source domain and $b_{i}$ represents item bias in the target domain.
- **MF**: This is the single-domain matrix factorization algorithm proposed in [2].
- **MF_MLP**: This is a cross-domain recommendation algorithm based on MF and MLP, which is proposed by [30]. In our experiment, for MF_MLP, the structure of the MLP is set as one-hidden layer, and the number of nodes in the hidden layer is set as 2M.

**Evaluation Metric.** We adopt the Root Mean Square Error (RMSE) to evaluate the prediction performance. It is defined as:

(16) $$RMSE=\sqrt{\sum_{y_{u_{i}}\in T^{\prime }}\frac{{\left(y_{u_{i}}-{\tilde{y}}_{u_{i}}\right)}^{2}}{|T^{\prime }|}},$$

where $T^{\prime }$ is the set of test ratings, $y_{u_{i}}$ denotes an observed rating in the test set, and ${\tilde{y}}_{u_{i}}$ represents the predictive value, $|T^{\prime }|$ is the number of test ratings.

### 5.2. Experimental Results

The experimental results in terms of RMSE on the Amazon dataset are presented in Table 2, where K represents the number of topics. For the sake of stringency of the experiments, we set different fractions for cold-start entities, namely, 20%, 50% and 70%. It can be seen that the performance of our proposed method is superior to other comparative methods.

As the proportion of cold-start users increases, the recommended performance decreases. The decline of single-domain recommendation method MF is the most obvious, while the performance of cross-domain recommendation method is relatively good, which also proves the effectiveness of knowledge transfer in cross-domain recommendation. The result of MF_MLP is better than MF, which also shows that the mapping function based on MLP is feasible in knowledge transfer. Compared with MF_MLP, the CDR-SAFM method proposed in this paper has been improved in terms of RMSE. When the number of topics K = 50, the mean square errors of our method are reduced by 0.0346, 0.0109 and 0.004 respectively. When the number of topics K = 100, the mean square errors are reduced by 0.0509, 0.0786 and 0.0829, respectively. These results demonstrate that the CDR-SAFM is more suitable for making recommendations to cold-start users compared to other cross-domain baseline methods, and also proves the effectiveness of our method in the cross-domain recommendation scenarios.

**Figure 5:**
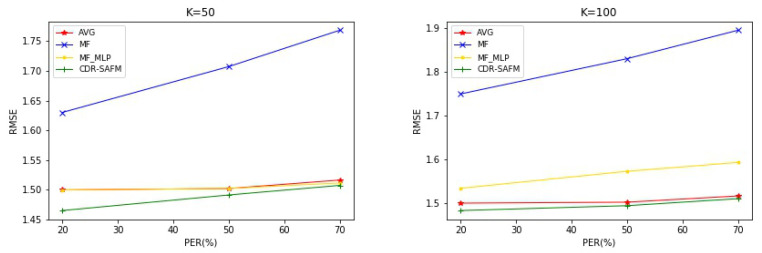
RMSE of the number of different topics.

In the topic model, the number of topics is very important. The number of topics directly affects the results of the experiment. However, the number of topics is not directly proportional to the results. As the number of topics increases, more computing costs will be required and there will be a risk of overfitting. In our method, we select the number of topics K as 50 and 100, and analyze the results under different number of topics. As shown in Figure 5, we tested RMSE with the number of topics K = 50 and K = 100 on the Electronics and Movies & TV datasets, respectively. We can see that the comparison results of AVG and MF_MLP are most obvious with the change of topic number. When the topic number K = 100, the difference between the two methods is more obvious. At the same time, we can see that when the number of topics K = 100, the CDR-SAFM method is relatively stable for different proportions of cold-start users in term of RMSE, which also proves the rationality of our method.

A cross-domain recommendation algorithm is an effective recommendation method. Its purpose is to transfer the knowledge in the source domain to the target domain, so as to improve the quality of recommendations and alleviate the cold start problem in the recommendation system. However, existing works on cross-domain algorithms mostly consider ratings, tags and text information such as reviews, but cannot use the sentiments implicated in the reviews efficiently. In this paper, we propose a cross-domain recommendation algorithm (CDR-SAFM) based on sentiment analysis and latent feature mapping by combining the sentiment information implicit in user reviews in different domains. The results of comparative experiments show that our method is effective. At the same time, it also proves that it is feasible for us to consider the implicit sentiment information in the reviews into the cross-domain recommendation method. However, there are still many deficiencies in our work. In this paper, we only validate our method on one dataset, and need to validate our method on more datasets in different fields. At the same time, there is a lot of space for data preprocessing and method improvement, which is also the problem we need to solve in future work.

## 6. Conclusions

In this paper, we address the cold-start problem in the recommendation process. We proposed a cross-domain recommendation algorithm based on sentiment analysis and latent feature mapping by combining the sentiment information implicit in user reviews in different domains. We first employed the latent feature model to project users in both source and target domains into two different feature spaces. Then, we learned an appropriate non-linear mapping function to capture the coordinate relationship across the two domains. To avoid mutual interference between different sentiment features during the process of knowledge transfer, we have learned three different types of sentiment mapping function, respectively based on three-way decision ideas, including positive, neutral and negative mapping functions. For a cold-start user in the target domain, we made recommendations by mapping a user’s sentiment features from the source domain to the target domain. Experimental results from a cross-domain recommendation scenario on the Amazon dataset demonstrate that the proposed framework can improve the quality of cross-domain recommendation. There are possible minimum biases in the rating process in relation to some factors as interests of raters. This work does not make a contribution to the bias. Developing an efficient method to preprocess the rating data is part of our planned future work.

## Figures and Tables

**Table 1:** Statistics of the Amazon dataset.

Datasets	Electronics	Movies & TV
Num. of users	192,403	123,960
Num. of Items	63,001	50,052
Num. of reviews	8,898,041	1,697,533

**Table 2:** Recommendation performance in terms of RMSE on the Amazon dataset.

K: the Number of Topics	Algorithms	20%	50%	70%
50	AVE	1.4998	1.5020	1.5163
50	MF	1.6300	1.7071	1.7682
50	MF_MLP	1.4996	1.5021	1.5113
50	CDR-SAFM	1.4650	1.4912	1.5073
100	AVE	1.4998	1.5020	1.5163
100	MF	1.7494	1.8300	1.8950
100	MF_MLP	1.5338	1.5726	1.5929
100	CDR-SAFM	1.4829	1.4940	1.5100
